# Assessment of muscle damage in patients with advanced Duchenne muscular dystrophy based on serum creatinine and the height-to-creatinine ratio

**DOI:** 10.3389/fneur.2026.1918003

**Published:** 2026-09-07

**Authors:** Binyan Wan, Huannan Li, Jie Shao, Tian Gao, Yongguang Shi, Congbo Sha, Xuen Yu

**Affiliations:** 1 Anhui University of Chinese Medicine, Hefei, Anhui, China; 2 Department of Neurology, Affiliated Hospital of the Institute of Neurology, Anhui University of Chinese Medicine, Hefei, Anhui, China

**Keywords:** creatine kinase, Duchenne muscular dystrophy, height-to-creatinine ratio, muscle magnetic resonance imaging, serum creatinine

## Abstract

**Background:**

Duchenne muscular dystrophy (DMD) is a fatal, X-linked neuromuscular disorder. This study investigated the correlation between serum markers and muscle damage in advanced DMD, retrospectively identified biochemical markers that reflect muscle damage in patients with advanced DMD, and provided a clinical basis for muscle assessment in advanced DMD.

**Methods:**

We enrolled 70 male patients with DMD who were treated at the Affiliated Hospital of the Institute of Neurology, Anhui University of Chinese Medicine, between January 2018 and December 2025. They were divided into an advanced group (≥12 years) and a non-advanced group (<12 years) based on age. We compared differences in serum creatinine (Scr), height-to-creatinine ratio, and creatine kinase (CK) levels between the two groups and used Spearman's rank correlation analysis to assess the association between these indicators and muscle damage.

**Results:**

The levels of Scr and CK in the advanced group were lower than those in the non-advanced group, while the height-to-creatinine ratio was higher (*P* < 0.05). The results of Spearman's rank correlation analysis showed that Scr levels in late-stage DMD were significantly correlated with magnetic resonance imaging (MRI) muscle damage scores (*r* = −0.522, *P* = 0.003), while the height-to-creatinine ratio was significantly positively correlated with MRI scores (*r* = 0.52, *P* = 0.003). Results of multiple linear regression analysis showed that Scr (*B* = −0.056, *P* < 0.001) and the height-to-creatinine ratio (*B* = −0.018, *P* = 0.047) were independent predictors of the degree of muscle damage in patients with advanced DMD. Scr exhibited the strongest effect (standardized β = −1.007), with an adjusted *R*^2^ of 0.375.

**Conclusion:**

Scr reflects changes in total skeletal muscle mass in patients with advanced DMD. In conjunction with the height-to-creatinine ratio, these parameters provide an assessment indicator for skeletal muscle damage.

## 1 Introduction

Duchenne muscular dystrophy (DMD) is a common X-linked monogenic neuromuscular disorder caused by pathogenic mutations in the DMD gene, which encodes dystrophin (1). The incidence is estimated at one in 6,500 live male births (2). In patients with DMD, muscle cells are gradually replaced by fatty and fibrotic tissue, manifesting as progressive muscle fiber necrosis and muscle atrophy (1). The severity of muscle damage in DMD progresses with age (3); subtle symptoms typically emerge by age two, with loss of independent ambulation occurring by age 12. Disease progression affects the respiratory and cardiac muscles, and patients typically die from respiratory or heart failure between the ages of 20 and 30. No curative therapy for DMD currently exists. The primary treatment comprises glucocorticoids, aimed at slowing disease progression and alleviating clinical symptoms (4). Accurate assessment of the extent of muscle damage is crucial for disease staging, monitoring treatment efficacy, and evaluating prognosis. Multiple studies have confirmed that muscle magnetic resonance imaging (MRI) can assess disease severity by evaluating the rate of fat infiltration or the extent of muscle damage (5, 6). However, MRI has limitations such as equipment dependency, time-consuming procedures, high costs, and poor patient cooperation, making it difficult for it to serve as a routine diagnostic tool in primary care hospitals or for long-term follow-up.

Serological markers enable rapid, non-invasive, and low-cost testing, making them suitable for bedside and continuous monitoring. Creatine kinase (CK) is an established biomarker of muscle injury (7); however, in patients with advanced disease, significant loss of muscle fibers leads to a marked decrease in CK levels, limiting its diagnostic value. Creatinine (Cr) is the end product of creatine catabolism in muscle. Under steady-state conditions, its production rate is relatively constant, and its production volume is proportional to total skeletal muscle mass (8). Existing investigations have shown that serum creatinine (Scr) can serve as a useful indicator for assessing the severity of muscle damage in DMD (9). However, Scr is confounded by height and body composition, and Cr alone cannot accurately reflect changes in muscle mass. This study evaluates the height-to-creatinine ratio as a corrective indicator and a supplementary measure for assessing the extent of muscle damage in DMD. It explores the correlation between Scr, the height-to-creatinine ratio, CK, and other serum markers with the degree of muscle damage as assessed by MRI and clarifies their value in evaluating muscle mass in advanced DMD. The aim is to establish a muscle volume assessment system based on serological indicators, providing a simple and reliable approach for the clinical evaluation and standardized management of DMD.

## 2 Materials and methods

### 2.1 Study design and participants

We retrospectively reviewed male patients who were treated at the Affiliated Hospital of the Institute of Neurology, Anhui University of Chinese Medicine, between January 2018 and December 2025 and who were diagnosed with DMD based on muscle biopsy or genetic testing. Inclusion criteria were as follows: (1) compliance with the diagnostic criteria for DMD outlined in the “Chinese Guidelines for the Diagnosis and Treatment of Duchenne Muscular Dystrophy” published by the Neurology Branch of the Chinese Medical Association; (2) age between two and 23 years; (3) completed serological testing. Exclusion criteria were as follows: concomitant renal disease, liver disease, malignant tumors, other types of myopathy, severe infections, and metabolic disorders.

Currently, there is no universally accepted definition of “late-stage” DMD; clinically, the loss of the ability to walk around the age of 12 is commonly used as a criterion (10). There is a certain correlation between age and muscle mass; after the age of 12, muscle fiber necrosis accelerates, and muscle volume decreases more rapidly in patients with DMD, marking the onset of the late-stage non-ambulatory phase (11). In this study, advanced DMD was defined as age ≥12 years, with the following groupings: advanced group: 30 cases (age ≥12 years); non-advanced group: 40 cases (age < 12 years).

### 2.2 Research method

#### 2.2.1 Data collection

We retrospectively collected general clinical data, serological test results, and MRI findings from 70 patients with DMD, compiled the data, and performed statistical analysis.

#### 2.2.2 MRI acquisition

Muscle MRI scans were performed on the bilateral thigh muscle groups. The imaging protocol included coronal T1-weighted imaging (T1WI), axial T2-weighted imaging (T2WI), and axial and coronal T2-weighted fat-suppressed sequences. Referring to the muscle MRI grading system established by Liu et al. (12), coronal T1-weighted imaging sequences were used to assess the degree of fat infiltration and injury in the thigh muscles, while T2-weighted and fat-suppressed sequences provided supplementary information on edema and inflammatory changes. All MRI scans were acquired in the axial plane at the mid-thigh level, defined as the midpoint between the greater trochanter and the femoral condyle, to ensure consistent slice positioning across patients. Quantification criteria were as follows: zero points: no injury; normal muscle morphology and signal intensity; one point: mild injury; slight reduction in muscle volume; homogeneous local signal intensity; two points: moderate injury; marked reduction in muscle volume; heterogeneous signal intensity; three points: severe injury; significant reduction in muscle volume; widespread fatty infiltration (high signal intensity on T1WI).

#### 2.2.3 Serological testing

We collected a fasting venous blood sample to measure Scr, CK, myoglobin (Mb), and other parameters. The height-to-creatinine ratio (cm/μmol/L) was calculated as height (cm)divided by Scr (μmol/L) to account for the influence of body size on Cr levels. Height data were extracted from electronic medical records, measured by trained nurses using a standardized protocol (supine length for non-ambulatory patients and standing height for ambulatory patients) at the time of hospital admission. Height was defined as the straight-line distance from the vertex to the heel, with the lower limbs gently extended to the maximum achievable position. All measurements were recorded to the nearest 0.1 cm.

### 2.3 Ethical requirements

This study complies with the 1964 Declaration of Helsinki and has been approved by the Ethics Committee of the Affiliated Hospital of the Institute of Neurology, Anhui University of Chinese Medicine, ethics approval number: 2022(26).

### 2.4 Statistical analysis

Data analysis was performed using IBM SPSS Statistics 26.0 (Armonk, New York, USA). Categorical data were expressed as the number of cases (%) and frequency and were analyzed using the chi-square test; continuous data that followed a normal distribution are expressed as mean ± standard deviation (x2 ± s), and comparisons between groups were performed using the independent samples *t*-test; non-normally distributed continuous variables were expressed as median (interquartile range) [M (Q1–Q3)], and the Mann–Whitney *U*-test was used for comparisons between groups. Spearman's rank correlation analysis was used to assess the correlation between serological markers and MRI atrophy scores, and multiple linear regression was used to evaluate the independent effects of each indicator on the degree of muscle atrophy. The significance level was set at α = 0.05.

## 3 Results

### 3.1 General information and clinical characteristics

Seventy patients with DMD were enrolled (Table 1). In the advanced group (30 patients), the age range was 12–23 years (mean 17.7 ± 3.385 years), the duration of illness was 13 years (range 10–15 years), and the mean height was 153 cm (range 150–155 cm); In the non-advanced group, comprising 40 patients, the age ranged from 2 to 11 years (7.57 ± 2.63), the duration of illness was 6 years (5.75, 7), and the height was 128 cm (124.5, 135.25). There were statistically significant differences between the two groups in age, duration of illness, height, and MRI-detected muscle damage (*P* < 0.05), while there were no statistically significant differences in hormone therapy between the two groups (*P* > 0.05).

**Table 1:** Baseline characteristics of the two groups.

Characteristic	Advanced group (*n* = 30)	Non-advanced group (*n* = 40)	*P-*value
Age, mean ± SD, years	17.7 ± 3.385	7.57 ± 2.63	< 0.001
Disease course, years	13 (10–15)	5 (3–6)	< 0.001
Height, cm	153 (150–158)	128 (123–135)	< 0.001
Hormone therapy, *n* (%)	30 (100)	38 (95)	0.214
Extent of MRI damage, *n* (%)	30 (100)	23 (57.5)	< 0.001
Level 0, *n* (%)	0	0	–
Level 1, *n* (%)	0	2 (8.7)	–
Level 2, *n* (%)	3 (10)	6 (26.1)	–
Level 3, *n* (%)	27 (90)	15 (65.2)	–

### 3.2 Comparison of differences in serological markers between the two patient groups

All measured serum markers, including muscle-related enzymes, cardiac biomarkers, and the height-to-creatinine ratio, showed significant differences between the two groups (*P* < 0.05), with Scr and other muscle-related markers being lower in the advanced group and the height-to-creatinine ratio being higher (Table 2).

**Table 2:** Serological parameters between the two patient groups.

Characteristic	Advanced group (*n* = 30)	Non-advanced group (*n* = 40)	*Z*	*P-*value
ALT	56 (39.5–70)	202 (141–310)	−6.724	0.000
AST	50 (41–65.5)	130 (112–213)	−6.422	0.000
Mb	403.8 (278.6–687)	639 (339.8–1045.5)	−2.079	0.038
LDH	258 (210.5–306.5)	783 (590–998)	−6.581	0.000
CK-MB	59 (48.5–89.5)	230 (172–380)	−6.541	0.000
Scr (μmol/L)	16 (10.5–19)	17 (13–20)	−2.16	0.031
height-to-creatinine ratio	10 (7.78–13.64)	7.24 (5.52–10.38)	−4.0	0.000
CK (U/L)	2542 (1949.5–3789.5)	9820 (5130–14085)	−5.827	0.000
Troponin I (ng/ml)	0.4 (0.25–0.75)	0.8 (0.5–1.38)	−2.943	0.003

### 3.3 Comparison of the extent of muscle injury on MRI between the two patient groups

**Figure 1:**
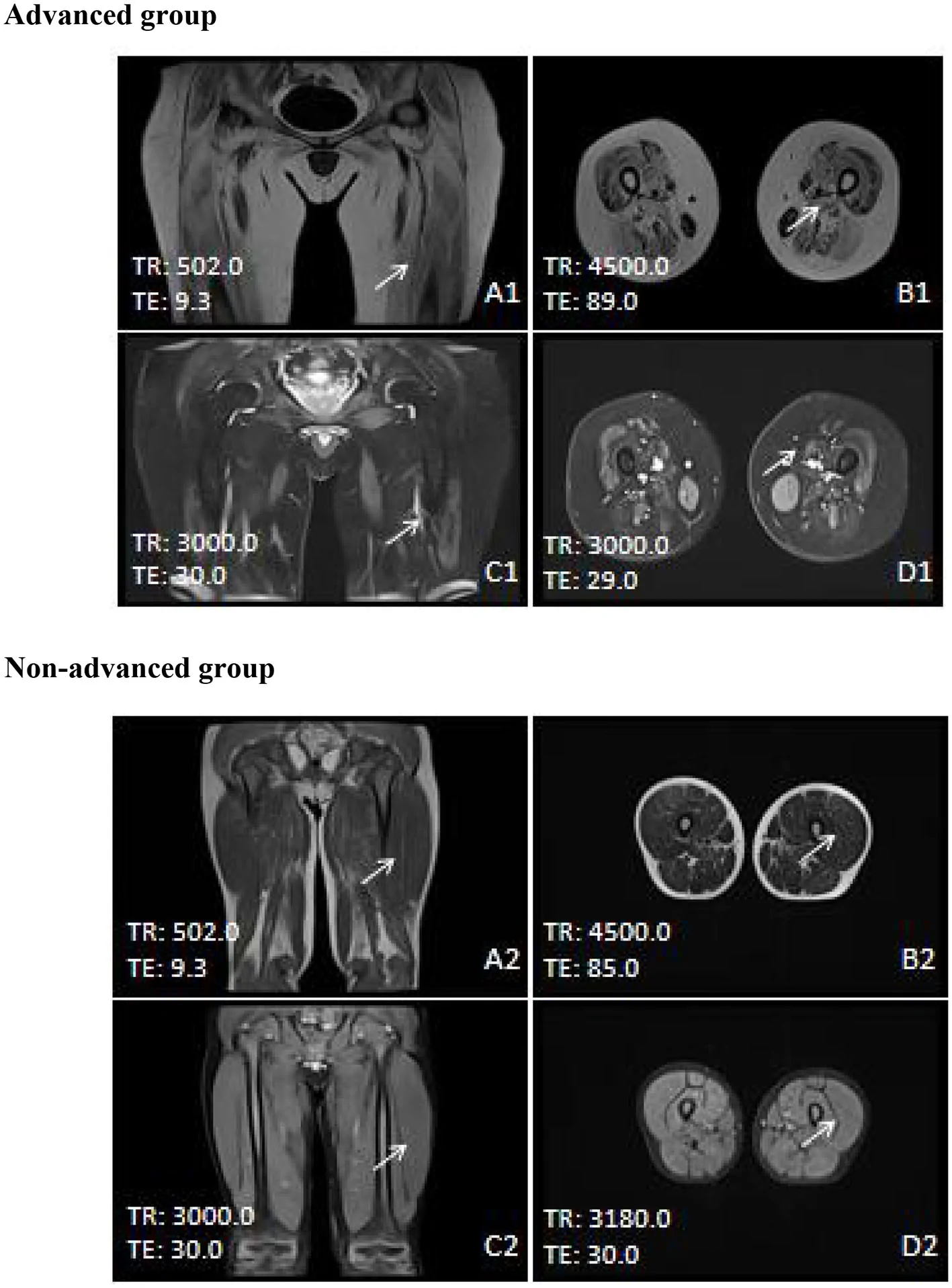
Representative muscle magnetic resonance imaging (MRI) findings in the advanced and non-advanced groups. Representative magnetic resonance images of the bilateral thigh muscles. **(A1–D1)** Advanced group (age ≥ 12 years). **(A2–D2)** Non-advanced group (age < 12 years). Images are presented in the following order: coronal T1-weighted imaging **(A1, A2)**, axial T2-weighted images **(B1, B2)**, coronal T2-weighted fat-suppressed imaging **(C1, C2)**, and axial T2-weighted fat-suppressed imaging **(D1, D2**). Coronal T1-weighted images demonstrate muscle anatomy, volume, and fatty infiltration, whereas T2-weighted and fat-suppressed sequences highlight increased tissue water content, including edema and inflammatory changes. All axial images were acquired at the mid-thigh level, defined as the midpoint between the greater trochanter and the femoral condyle. Arrows indicate regions of muscle damage. DMD, Duchenne muscular dystrophy.

Of the 53 patients with evaluable MRI datasets (Figure 1), 90% (27/30) of the advanced group had severe lesions, whereas 10% (3/30) had moderate lesions; in the non-advanced group, 65.2% (15/23) had severe lesions, 26.1% (6/23) had moderate lesions, and 8.7% (2/23) had mild lesions. The proportion of severe muscle damage was higher in the advanced group than in the non-advanced group, suggesting that muscle loss was more severe in patients with advanced DMD; however, as noted in the introduction, MRI has certain limitations in this population-particularly in patients with advanced DMD, who often have difficulty maintaining the required position due to joint contractures and severe muscle weakness, leading to poor image quality or inability to complete the examination. These practical constraints limit the routine use of MRI for longitudinal monitoring in advanced DMD and underscore the need for more accessible serological assessment tools.

### 3.4 Analysis of the correlation between serological markers and the severity of muscle injury on MRI within the two patient groups

Among the 30 patients with advanced DMD who had complete MRI data, Spearman's correlation analysis revealed (Table 3) that Scr levels in the advanced group were significantly negatively correlated with the extent of muscle damage on MRI (*r* = −0.522, *P* = 0.003); the height-to-creatinine ratio was significantly positively correlated with muscle damage on MRI (*r* = 0.52, *P* = 0.003).

**Table 3:** Correlation analysis between serological markers and the severity of muscle injury as assessed by MRI in the two groups.

Characteristic	Advanced group		Non-advanced group	
	*R*	*P-*value	*r*	*P-*value
SCr	−0.522	0.003	0.338	0.114
CK	−0.173	0.36	0.342	0.111
CK-MB	−0.224	0.244	−0.24	0.27
LDH	−0.019	0.92	−0.38	0.074
Mb	−0.149	0.441	−0.256	0.238
Troponin I	0.054	0.78	−0.114	0.613
AST	−0.334	0.071	−0.235	0.28
Uric acid	−0.135	0.478	−0.133	0.544
Lactic acid	−0.237	0.206	−0.13	0.566
height-to-creatinine ratio	0.52	0.003	−0.294	0.174

Only 23 patients in the advanced group completed the magnetic resonance imaging (MRI) scan.

### 3.5 Regression analysis between serological markers and MRI-measured severity of muscle damage in the advanced group

To further control for the influence of confounding factors, a multiple regression model was constructed (Table 4) for patients with advanced DMD, using MRI muscle damage scores as the dependent variable and height-to-creatinine ratio, Scr, and *CK* as independent variables.

**Table 4:** Regression analysis of serum creatinine and height-to-creatinine ratio on the extent of muscle damage as assessed by magnetic resonance imaging (MRI) in the advanced group.

Characteristic	Non-standardization		Standardization	*t*	*P*	Collinearity diagnosis	
	*B*	Standard error	Beta			*VIF*	Tolerance
(Constant)	3.880	0.288	–	13.462	0.000	–	–
CK	2.116E-5	0.000	0.138	0.858	0.399	1.198	0.835
height-to-creatinine ratio	−0.018	0.009	−0.487	−2.083	0.047	2.537	0.394
Scr	−0.056	0.013	−1.007	−4.178	0.000	2.696	0.371
*R* ^2^				0.440			
Adjusted *R*^2^				0.375			
*F*			*F* = 6.798, *P* = 0.002				
*D*–*W* value				1.524			

In the regression analysis of Scr on the extent of muscle injury as measured by MRI, the variance inflation factor (VIF) was less than five, indicating no multicollinearity issues. The regression model was significant (*F* = 6.798, *P* = 0.002), with an adjusted *R*^2^ of 0.375, meaning the independent variables explained 37.5% of the variation in the dependent variable. In the significance test of regression coefficients, both Scr (*B* = −0.056, *P* < 0.001) and the height-to-creatinine ratio (*B* = −0.018, *P* = 0.047) had a significant effect on the severity of late-stage MRI muscle injury. Based on the standardized regression coefficients, Scr showed a stronger influence on MRI muscle injury severity than the height-to-creatinine ratio.

## 4 Discussion

DMD is the most severe form of muscular dystrophy and is caused by a deletion or mutation in the DMD gene located at Xp21.1-21.3 on the X chromosome (13). Clinically, it is characterized by progressive muscle atrophy, loss of ambulation, and impaired cardiopulmonary function; therefore, accurate assessment of muscle mass is essential for clinical management. Analysis of serum markers in 70 patients revealed that Scr levels were significantly reduced in patients with advanced disease and showed a significant negative correlation with the extent of muscle damage as indicated by MRI, suggesting that Scr can reflect changes in total skeletal muscle mass. Additionally, the height-to-creatinine ratio effectively eliminates the confounding effects of height and body build and shows a significant positive correlation with the extent of muscle damage, making it a suitable corrective indicator for Scr.

As DMD progresses, muscle fibers undergo progressive necrosis, muscle volume decreases, Cr production declines, and serum Cr levels drop, which is consistent with the present findings. Previous studies have demonstrated that Cr excretion gradually decreases with muscle atrophy (14), 24-h urinary Cr excretion can serve as a reliable indicator of muscle content (15), and Scr is highly correlated with urinary Cr excretion (16). A study by Zhang et al. (9) involving 148 children with DMD demonstrated a strong negative correlation between Scr and the Vignos functional score (a widely used scale for assessing lower extremity functional status in DMD, ranging from one [ambulatory] to 10 [wheelchair-dependent]). Wang et al. (17) demonstrated that Cr levels can be used to assess disease progression in muscular dystrophy. Nambu et al. (8) demonstrated that Cr can serve not only as an independent indicator of muscle mass but also as a denominator to correct for other muscle-specific proteins, thereby eliminating interindividual variations in total muscle mass. Groothof et al. (18) provided further evidence of the direct association between Cr levels and muscle mass from the perspective of renal function assessment. Building upon these prior observations, the present study further examined the association between Scr and the extent of muscle damage as assessed by MRI, showing that Scr in patients in the advanced group was significantly negatively correlated with MRI muscle damage scores (*r* = −0.522, *P* = 0.003), thereby validating the reliability of Scr as an indicator of muscle damage from an imaging perspective. Multivariate linear regression analysis further demonstrated that Scr is an independent factor influencing muscle damage in the advanced group. Lower Scr levels correlate with more severe muscle damage as shown by MRI, reflecting the extent of damage to total skeletal muscle mass in patients with the advanced group.

In patients in the advanced group, long-term use of corticosteroids, loss of ambulation, and muscle atrophy accompanied by adipose tissue infiltration can lead to variations in height and body composition that may interfere with Scr assessment results. The 2018 International Guidelines for the Diagnosis and Management of DMD state that long-term glucocorticoid therapy is the standard treatment for DMD, but it can lead to complications such as osteoporosis and growth retardation (19). A Slovenian national cohort study demonstrated that the vast majority of DMD patients exhibit reduced stature following steroid therapy (20). A decrease in Scr levels alone cannot distinguish between reduced muscle mass and differences in body build. Zhang et al. (9) found that after adjusting for height, the value of Scr in assessing muscle damage was not diminished; rather, it more accurately reflected changes in muscle mass. Therefore, this study employs the height-to-creatinine ratio to correct body build interference, thereby enhancing assessment accuracy and providing a more reliable clinical indicator. The necessity of this approach is supported by multiple studies; a large-scale analysis by Park et al. (21) involving numerous adults confirmed a significant association between height and creatinine-related indicators. van Dam et al. (22) validated the feasibility of using the serum creatinine-to-height ratio to assess muscle metabolic indicators in 600 overweight children. Uemura et al. (23) confirmed that Scr levels in healthy children are linearly and positively correlated with height. Collectively, these studies indicate that it is necessary to account for the confounding effects of height when analyzing Cr levels in DMD patients. This study shows that the height-to-creatinine ratio in patients in the advanced group is significantly positively correlated with MRI-measured muscle damage (*r* = 0.520, *P* = 0.003). Regression analysis indicates that the height-to-creatinine ratio, adjusted for height, can independently predict the extent of muscle damage, further confirming that correcting for body size confounders improves the accuracy of Scr assessment. Univariate analysis indicated a positive correlation between the height-to-creatinine ratio and the degree of muscle damage on MRI (*r* = 0.520), whereas in multivariate regression, the coefficient for this indicator was negative (*B* = −0.018), indicating that, after adjusting for Scr and other factors, a higher height-to-creatinine ratio was actually associated with less severe muscle damage on MRI. This is consistent with the interpretation that a higher ratio reflects better-preserved muscle mass. The apparent reversal in direction between the univariate and multivariate analyses is likely due to the limited height variation in patients with advanced DMD, which does not diminish the clinical utility of this indicator.

CK, a traditional marker of skeletal muscle damage, is significantly elevated in the early stages of DMD and can aid in disease diagnosis (24). However, in the late stages of DMD, extensive muscle fiber necrosis and a significant reduction in functional muscle volume lead to a marked decrease in CK levels. A longitudinal study by Zygmunt et al. (25) involving 555 patients with DMD demonstrated that CK levels decline continuously with age and are positively correlated with the deterioration of motor function. In a serum proteomics study of mdx-4cv mice, Murphy et al. (26) noted that changes in the concentrations of traditional serum markers such as CK are inconsistent across acute and chronic disease phases and lack high specificity, which limits their reliability in the assessment of chronic DMD. Meng et al. (27) pointed out that CK has fundamental limitations as a biomarker; its levels are unrelated to the severity of DMD and therefore cannot specifically reflect the extent of muscle damage. The multiple linear regression analysis in this study showed that CK is not an independent factor influencing muscle damage in patients in the advanced group (*P* > 0.05), which is consistent with the conclusions of the aforementioned studies. In summary, CK should not be used alone as an independent indicator for assessing muscle mass loss in advanced DMD, but it can serve as an auxiliary indicator.

Muscle MRI is currently an important imaging method for assessing the severity of DMD. However, in clinical practice, its widespread use is often limited in patients with advanced disease due to difficulties in patient cooperation and equipment constraints. The results of this study confirm that Scr combined with the height-to-creatinine ratio can serve as a rapid bedside assessment tool, helping to reduce healthcare costs and improve long-term follow-up compliance, and that it is suitable for long-term monitoring in primary care settings and at home.

## 5 Conclusion

In summary, Scr levels in patients with advanced DMD decrease as muscle damage worsens, and the height-to-creatinine ratio effectively corrects for interference caused by body size; both are independent predictors of the extent of muscle damage in advanced DMD. The combination of Scr and the height-to-creatinine ratio enables non-invasive, low-cost, reproducible assessment of muscle damage. It holds significant clinical value for disease monitoring and long-term follow-up in patients with advanced DMD, laying the foundation for the establishment of a standardized assessment system. This study has the following limitations: (1) As a retrospective study, data were derived from clinical case records, and some indicators had minor missing values, which may affect the accuracy of the results. (2) As a cross-sectional study, it cannot reveal the dynamic changes in serum markers over the course of the disease; longitudinal cohort studies are needed to address this. (3) A comprehensive systematic assessment of renal function was not conducted, so the influence of renal function on Cr levels cannot be completely ruled out. (4) Finally, in the absence of a healthy control group, the analysis was conducted exclusively using data from patients with DMD. Prospective studies with healthy participants are needed to corroborate our findings.

## Data Availability

The datasets presented in this study can be found in online repositories. The names of the repository/repositories and accession number(s) can be found in the article/supplementary material.
